# Effect of Ripening in Brine and in a Vacuum on Protein, Fatty Acid and Mineral Profiles, and Antioxidant Potential of Reduced-Fat White Cheese

**DOI:** 10.17113/ftb.59.01.21.6891

**Published:** 2021-03

**Authors:** Miroljub Barać, Zlatan Sarić, Tanja Vučić, Ivana Sredović Ignjatović, Danijel Milinčić, Bojana Špirović Trifunović, Milenko Smiljanić

**Affiliations:** 1 University of Belgrade, Faculty of Agriculture, Nemanjina 6, 11080 Zemun, Serbia; 2 University of Sarajevo, Faculty of Agriculture and Food Sciences, Zmaja od Bosne 8, 71000 Sarajevo, Bosnia and Herzegovina; 3 University of East Sarajevo, Faculty of Technology, Karakaj 34A, 75400 Zvornik, Bosnia and Herzegovina

**Keywords:** reduced-fat white cheese, proteolysis, fatty acids, mineral profile, antioxidant properties

## Abstract

**Research background:**

Numerous factors affect the ripening of reduced-fat white cheese. The aim of this study is to investigate the influence of ripening environment (brine or vacuum plastic bags without brine) on the chemical composition, protein, fatty acid profile and mineral content as well as antioxidant properties of industrially produced reduced-fat white cheese.

**Experimental approach:**

A low-fat white cheese was manufactured on an industrial scale from milk that remained after the production of kajmak and ripened for 60 days at 4 °C after packaging in a polystyrene container with brine containing 6% salt or in vacuum-sealed polyethylene bags. The influence of ripening environment on proteolysis was monitored by the change of soluble nitrogen fractions as well as by sodium dodecyl sulphate–polyacrylamide gel electrophoresis of tris(hydroxymethyl) aminomethane-HCl extracts of cheese proteins under non-reducing conditions and water-soluble fractions under reducing conditions. An effect that ripening environment had on fatty acid and mineral content was also monitored. The change of antioxidant potential of the investigated cheese during ripening led to the change of iron(II) chelating ability, reducing power and free-radical scavenging activity.

**Results and conclusions:**

The ripening environment differently affected proteolysis, fatty acid composition, mineral profile and antioxidant properties of reduced-fat white cheese. White cheese ripened in brine had more intensive proteolytic changes than the cheese ripened in a vacuum, but also more intensive diffusion processes, especially between the 40th and 60th day of ripening. The brine-ripened cheese had higher values of water-soluble nitrogen content, but lower contents of trichloroacetic acid-soluble and phosphotungstic acid-soluble nitrogen than the vacuum-ripened cheese. Cheese ripened in brine had a lower content of almost all investigated macro- and microelements. After 60 days of ripening, in cheese ripened in brine only myristic (C14:0) and palmitic acid (C16:0) were detected, whereas in the vacuum-ripened cheese C10:0-C16:0 fatty acids dominated. Vacuum-ripened reduced-fat cheese had more favourable reducing power, while white brined reduced-fat cheese had better radical scavenging activity and iron(II) chelating activity.

**Novelty and scientific contribution:**

These results suggest significant influence of ripening conditions (immersion in brine or in vacuum-sealed polyethylene bags) on nutritive and functional properties of reduced-fat white cheese. Ripening in a vacuum has become a useful method for obtaining high-value reduced-fat white cheese.

## INTRODUCTION

In recent years, there has been increased interest in the industrial production of kajmak, a traditional product of Southeast European countries that can be described as a skin-like layer that forms during cooling of previously high-heat treated milk. The production of kajmak is based on the aggregation of milk fat and the inclusion of a part of milk proteins. Milk that remains after the kajmak production is used mainly for the production of reduced- or low-fat white brined cheese. However, residual milk has specific properties that can affect cheese characteristics. Residual milk is characterized by a low content of fat and fat-soluble substances, as well as slightly lower protein content than the raw milk. The low fat content of milk can affect the composition, ripening biochemistry, microstructure, organoleptic/sensory characteristics and yield of the cheese (*1*). In addition, during the production of kajmak, milk is subjected to more severe thermal processing (at 90 °C for up to 60 min) than typical pasteurization and slow cooling (up to 24 h). It is known that high-heat treatment induces whey protein denaturation, especially α-lactalbumin (α-La) and β-lactoglobulin (β-Lg) and the formation of whey protein‒casein complexes (*2*). The presence of high amount of these complexes increases the rennet clotting time of milk, whereas the incorporation of micellar whey protein-casein complexes into the cheese curd reduces syneresis, increases water-binding ability (*3*) and affects proteolysis (*4*). Furthermore, a long period of milk fat separation provides proteolytic activity of thermostable enzyme plasmin and activity of bacteria that originate from the environment. This may additionally affect the coagulation of milk and the cheese ripening process.

White cheese made from milk that remains after the production of kajmak is usually ripened and marketed in brine, while in well-organized dairy plants after ripening in brine cheese is vacuum packaged in polyethylene bags and then distributed. Ripening in brine can negatively affect the nutritional value of reduced-fat white cheese. Due to the diffusion processes that occur in brine, these types of cheese can lose part of the nitrogenous substances, macro- and microelements and can have a relatively high NaCl content. Thus, from a practical and hygienic aspect, ripening without brine in vacuum-sealed plastic bags could be more convenient. Besides the low fat content, cheese ripened in a vacuum would also have a low level of salt and would be suitable for those who avoid consuming white cheese because of its high salt content. Furthermore, ripening in a vacuum instead of brine would avoid the loss of a part of low-molecular-mass peptides. Since these peptides are mainly responsible for cheese bioactivity, this could contribute to higher nutritive value. Finally, vacuum-sealed package is more hygienic, it prolongs the shelf-life and makes this type of cheese more suitable for large-scale market distribution and broader consumption than the traditionally ripened in brine.

Although the biochemistry of ripening of different reduced-fat cheese varieties was the object of several studies (*1*, *5*-*8*), to the best of the authors’ knowledge, there are no studies related to the ripening of reduced-fat white cheese made from milk that remains after kajmak production. Also, there is no study about the effect of ripening in a vacuum on the composition and properties of reduced-fat white cheese.

This study aims to compare the effect of ripening in brine or vacuum plastic bags without brine on the chemical composition, protein and fatty acid profiles, mineral content, as well as antioxidant properties of industrially produced reduced-fat white cheese. This can contribute to a better understanding of the ripening of reduced-fat cheese. Also, the obtained results can contribute to the modification of existing technologies in the development of reduced-fat cheese with higher nutritional value.

## MATERIALS AND METHODS

### Cheese making

**Fig. S1:**
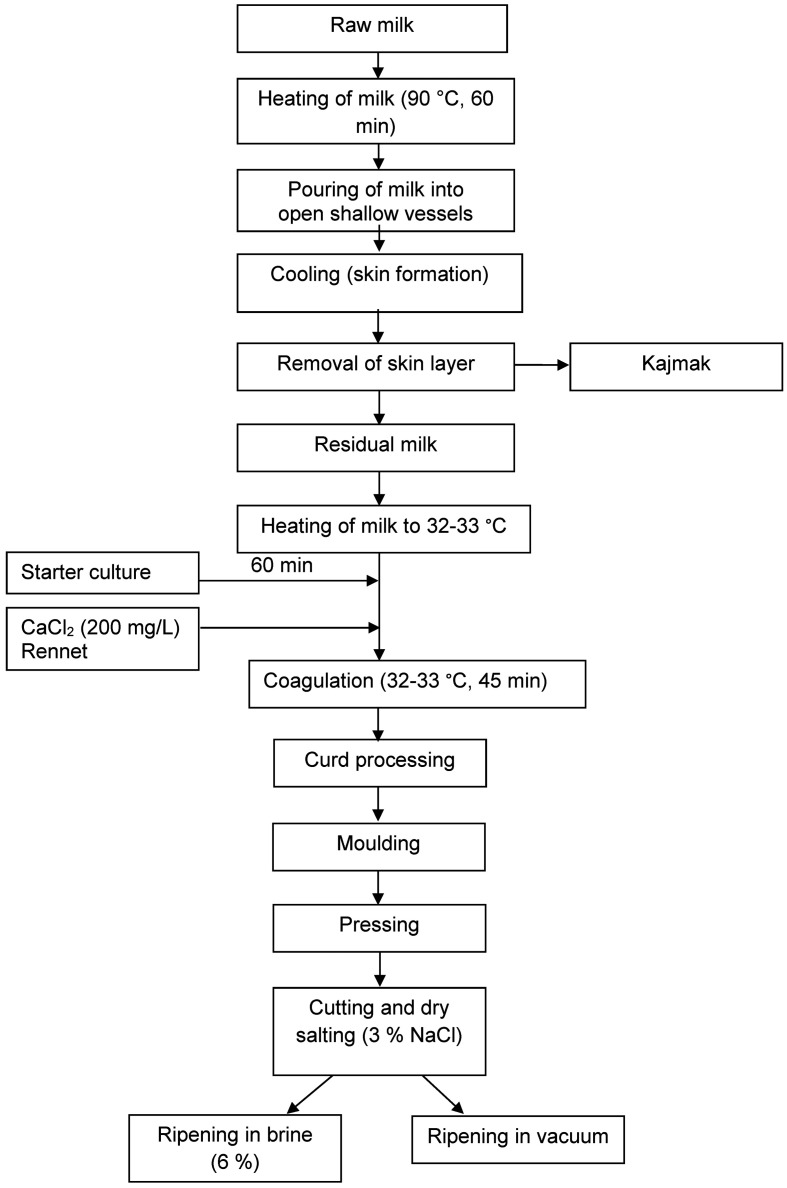
Flow chart of reduced-fat white cheese production

The Serbian reduced-fat white cheese samples were manufactured on an industrial scale in a local dairy Spasojevic d.o.o (Bajina Basta, Serbia), from cow’s milk that remained after the production of kajmak using a kajmak production device patented by Marković and Maćej (*9*). Cheese was made on three consecutive days in three replicates. The average content of fat and protein of the raw milk used was 3.88 and 3.66%, respectively, and the average pH was 6.64. The pretreatment of the milk and the protocol for the reduced-fat cheese production are shown in Fig. S1. The average content of fat and protein of the residual milk was 1.07 and 3.37%, respectively, and the average pH was 6.10. The milk was tempered at 32-33 °C and commercial starter culture methotrexate washed cells (MTX WC; Dalton Biotecnologie, Spoltore, Italy) was added. The composition of the starter culture was: *Lactococcus lactis* ssp. *lactis*, *Lactococcus lactis* ssp. *cremoris*, *Streptococcus salivarius* ssp. *thermophilus*, *Lactobacillus delbrueckii* ssp. *bulgaricus* and *Lactobacillus helveticus*. After 1 h, CaCl_2_ (200 mg/L, as 20% solution) and commercial rennet (90 mg/L; Maxiren, DSM Food Specialties BV, Delft City, The Netherlands) were added. Curd was formed within 45 min at 32–33 °C. Next, the curd was cut crossways in cubes of 1 cm^3^ and tempered at 37-38 ºC for about 40 min. Then, it was gently agitated for 15 min to facilitate whey expulsion and carefully transferred into the mould (80 cm×50 cm×20 cm). After that, the curd was left for 30 min to settle under the pressure of its own weight, and then pressed for 1 h at 50 kPa and another hour at 100 kPa. After that, the cheese curd was cut into pieces of 10 cm×10 cm×4 cm, dry salted with *w*(NaCl)=3.0% and left overnight. The next day, half of the cheese slices of each batch were placed into plastic containers (30 cm×25 cm×15 cm), covered with *w*(NaCl)=6% brine and closed with a lid. The other half of the slices was packed (each slice individually) into vacuum-sealed plastic bags. The cheese samples were ripened at 4 °C for 3 days in a dairy plant, transported to the laboratory and then stored for 60 days at the same temperature and 70% humidity in the ripening room. The cheese was sampled at 3, 20, 40 and 60 days of ripening.

### Chemical analysis

Prior to analysis, the cheese samples (whole slices) were ground to achieve uniformity. The chemical composition of the cheese samples was determined using the following methods: dry matter by the standard drying method at (102±2) °C (*10*), fat content according to the gravimetric method (*11*), nitrogen content by the AOAC method (*12*) and NaCl content by the Volhard method (*13*). The protein content was calculated as the nitrogen content multiplied by nitrogen conversion factor 6.38. Using these parameters fat and protein in dry matter, moisture in non-fat solids and salt in moisture were calculated and expressed as mass fraction in %. The pH of the cheese samples was measured using a pH meter (Consort, Tumhout, Belgium) (*14*).

### Proteolysis assessment

Proteolysis was monitored by measuring the contents of water-soluble nitrogen, 12% trichloroacetic acid-soluble nitrogen and 5% phosphotungstic acid-soluble nitrogen according to Kuchroo and Fox (*15*). These parameters were expressed as a percentage of total nitrogen. Besides these parameters, sodium dodecyl sulphate–polyacrylamide gel electrophoresis (SDS-PAGE) of tris(hydroxymethyl) aminomethane-HCl (Tris-HCl) extracts of cheese proteins under non-reducing conditions and water-soluble fractions under reducing conditions, according to the Fling and Gregerson method (*16*) was done. Electrophoresis was performed on 5% (*m*/*V*) stacking and 12.5% (*m*/*V*) resolving gel (gel electrophoresis apparatus model LKB-2001; LKB, Uppsala, Sweden). The water-soluble fraction was extracted as described by Kuchroo and Fox (*15*), and diluted with Tris-HCl buffer (0.055 M Tris-HCl, pH=6.8, 2% SDS, 5% β-mercaptoethanol (*V*/*V*), 7% glycerol, 0.0025% bromophenol blue). Tris-HCl extracts of cheese proteins were prepared using the same buffer but without β-mercaptoethanol. After filtration, the cheese extracts were diluted to 2 mg/mL. Low-molecular-mass calibration kit (Pharmacia, Uppsala, Sweden) was used to estimate molecular masses of the polypeptides in water-soluble fraction. A mixture of the major milk proteins (α_s_-, β- and κ-casein, α-lactalbumin and β-lactoglobulin; Sigma-Aldrich, Merck, St Louis, MO, USA) prepared under the same non-reducing conditions was used for identification of cheese protein bands. The scanned gels were analyzed by SigmaGel software v. 1.1 (*17*). Caseins and polypeptides were quantitatively determined by integration of peaksignals. Intensity of casein bands was quantified from the gel on which 25 μL of the samples were applied. Each pattern was analyzed in triplicate. Residual content of identified caseins was expressed as a mass fraction of their initial mass of 3-day-old cheese.

### Analysis of fatty acids

Prior to fatty acid and mineral content analyses, the cheese samples were frozen at -80 °C and lyophilized (at -44 °C and 9.7 Pa overnight) using Labconco freeze dryer (Labconco Corp., Kansas City, MO, USA). The lipid compounds were extracted from lyophilized cheese samples using hexane and transferred into fatty acid methyl esters (FAMEs) as we reported in our previous study (*18*). FAMEs were separated using capillary gas chromatography with flame ionization detector (model 6890; Agilent Technologies, Santa Clara, CA, USA) equipped with split/splitless injector and SP-2560 (length 100 m, i.d. 0.25 mm, film thickness 0.20 μm; Supelco, Bellefonte, PA, USA). The obtained chromatographic peaks were identified using Supelco 37 Component FAME mix standard. Fatty acid content was calculated in mg/g lipids and expressed as mass fraction (in %) of total fatty acids.

### Mineral profile

Lyophilized cheese samples were digested in a microwave digestion system (microwave oven Berghof, Speedwave, Berghof, Germany) using concentrated nitric acid and hydrogen peroxide. Macroelements (Ca, Mg, Na and K) were determined by flame atomic absorption spectroscopy (200 Series AA; Agilent Technologies, Inc.), whereas the microelements (Fe, Zn, Cu and Mn) were determined by inductively coupled plasma-mass spectrometry (ICP-MS model iCAP Q; Thermo Scientific, Oxford, UK). A multi-element stock solution XXI for MS (MES-21-5; AccuStandard, New Haven, CT, USA) containing 10 µg/mL of each element was used for the preparation of working standard solutions for the ICP-MS measurements by serial dilutions. Individual single-element stock solutions containing 1000 mg/L Ca, Mg, Na and K were supplied by AccuStandard. Murphy-Riley method, a dry ashing colourimetric method (*19*) was used to determine the content of phosphorus. This method is based on the interaction between phosphorus and ammonium molybdate and formation of blue phosphomolybdate complexes. The absorbance was measured at 700 nm using Shimadzu 1900i spectrophotometer (Shimadzu Corporation, Kyoto, Japan). The mass fraction of phosphorus in cheese was calculated using standard calibration curve for phosphorus, and expressed in mg/100 g.

### Antioxidant properties of cheese

The iron(II) chelating ability and the reducing power of cheese were measured and expressed as previously described in detail (*4*). The chelating activity was calculated as follows:


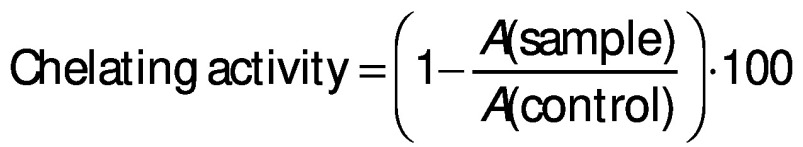


The reducing power of cheese was expressed as the absorbance of the reaction mixture measured at 700 nm. A higher absorbance indicates a greater reducing power.

Free radical scavenging activity was evaluated using a previously published method (*20*). Briefly, the stock solution (7 mM aqueous solution of ABTS (2,2-azino-bis(3-ethil-benothiazoline-6-sulphonic acid) with 2.45 mM potassium persulfate) was allowed to stand in the dark for 16 h. The working solution of ABTS was prepared by diluting the stock solution with methanol to obtain an absorbance between 0.7 and 0.8 at 734 nm. Thereafter, 10 mg of cheese were mixed with 1 mL of ABTS working solution and intensively stirred. After 7 min, samples were centrifuged for 5 min at 17 000×*g*, and the absorbance of the supernatants was measured at 734 nm. Percentage of quenched radicals for standard and samples was calculated as:


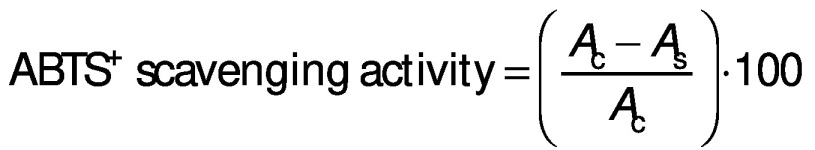


where *A*c is the absorbance of ABTS working solution, *A*s is the absorbance of sample or standard solution mixed with ABTS working solution. Butylated hydroxytoluene (BHT) solutions ranging from 10 to 100 μg/mL were used to prepare a calibration curve. The free radical scavenging activity was expressed as the percentage of inhibition as well as the BHT equivalent in μg/mL.

### Statistical analysis

Data were subjected to one-way analysis of variance (ANOVA) with IBM-SPSS v. 20 software (*21*). The analyses were performed in duplicate at each sampling time for each cheese variant. All measurements were performed in triplicate. Mean values were compared with Tukey’s test at p<0.05. Interactions between variables were evaluated and interpreted *via* linear correlation coefficients using Pearson’s correlation.

## RESULTS AND DISCUSSION

### Compositional analysis

Table 1 shows the chemical composition of the cheese samples analyzed during 60 days of ripening. Dry matter and protein in dry matter (PDM) mass fractions were significantly (p<0.05) lower in cheese ripened in brine than in the cheese samples ripened in a vacuum. This can be attributed to the water absorption from the brine, especially during the first days of the second stage of ripening in the cold storage because: (*i*) at low temperature the casein-bound calcium tends to be soluble, thus increasing casein hydration, (*ii*) the peptides coming from proteolysis bind water (*22*), and (*iii*) the heat-induced incorporation of hydrophilic whey proteins which increases the ability of a cheese matrix to bind water tightly (*23*).

**Table 1:** The chemical composition and pH of reduced-fat white cheese

*t*(ripening*)*/day	Cheese ripened in a vacuum							
*w*(DM)/%	*w*(FDM)/(%)	*w*(PDM)/%	*w*(MNFS)/%	pH	*w*(NaCl)_cheese_/%	*w*(NaCl)_moisture_/%	Acidity/°SH
3	(43.0±0.7)^a^	(29.1±0.6)^b,c^	(55.4±0.6)^a^	(65.2±0.7)^e^	(5.06±0.01)^c^	(1.56±0.04)^d^	2.66^e^	(82.9±0.6)^d^
20	(42.5±0.7)^a^	(30.0±0.7)^b^	(55.9±0.7)^a^	(65.9±0.6)^e^	(5.05±0.00)^c^	(1.67±0.04)^c^	2.82^d^	(90.3±0.5)^c^
40	(41.1±0.6)^a^	(28.3±0.9)^b,c^	(54.4±1.0)^a,b^	(66.7±0.4)^e^	(4.96±0.00)^d^	(1.62±0.05)^c^	2.67^e^	(95.3±0.3)^b^
60	(41.5±0.3)^a^	(28.9±1.6)^b,c^	(55.6±0.7)^a^	(66.5±1.1)^e^	(4.97±0.00)^d^	(1.53±0.04)^d^	2.61^e^	(98.8±0.3)^a^
Cheese ripened in brine								
3	(39.2±0.3)^b^	(27.4±0.9)^c^	(53.9±0.4)^b^	(68.1±0.3)^d^	(5.42±0.00)^a^	(2.30±0.06)^b^	3.65^b^	(75.5±0.5)^f^
20	(37.7±0.2)^c^	(27.9±0.8)^c^	(52.5±0.4)^c^	(69.6±0.5)^c^	(5.33±0.02)^b^	(2.47±0.15)^a,b^	3.81^a^	(78.8±0.6)^e^
40	(35.68±0.08)^d^	(29.43±0.07)^b^	(51.2±0.2)^d^	(71.87±0.09)^b^	(5.34±0.02)^b^	(2.54±0.04)^a^	3.80 ^a^	(70.5±0.2)^g^
60	(28.0±0.1)^e^	(33.9±0.1)^a^	(48.8±0.4)^e^	(79.5±0.1)^a^	(5.37±0.0)^b^	(2.39±0.02)^b^	3.21^c^	(50.5±0.3)^h^

DM=dry matter, FDM=fat in dry matter, MNFS=moisture in non-fat solid, PDM=protein in dry matter. Data within the same column marked with different lowercase letters are significantly different at p<0.05

The lower values of PDM in cheese samples treated in brine than in vacuum are probably the result of the diffusion of low-molecular-mass nitrogen compounds which formed during ripening in brine. In fact, dry matter and protein content in the cheese samples in brine decreased throughout the 60-day ripening. Thus, strong negative correlations between the time of ripening and dry matter  (-0.941, p<0.05) as well as between the time of ripening and protein content (-0.993, p<0.05) in these samples were found. This agrees well with the increase of moisture content in non-fat solids (MNFS) (Table 1). The most intensive decrease of dry matter mass fraction was observed between 40-60 days of ripening. It seems that this period of ripening is crucial for biochemical and sensory characteristics of reduced-fat cheese ripened in brine. In contrast to the cheese in brine, no significant (p<0.05) changes of dry matter, PDM and MNFS were found within 60 days in the cheese ripened in vacuum-sealed plastic bags.

From the results presented in Table 1, it is evident that cheese in brine had significantly (p<0.05) higher values of MNFS than vacuum-sealed cheese. Namely, MNFS of cheese in brine during ripening increased continually from 68.08 to 79.53%, whereas no significant changes were noticed in the vacuum-sealed cheese. Since a small mass fraction increase in MNFS (2–4%) leads to a relatively large increase in the available water, which in turn leads to increases in the activity of enzymes and microorganisms (*24*), a higher level of proteolytic products in brined than in vacuum-sealed cheese can be expected.

Reduced-fat cheese generally had the similar fat in dry matter (FDM) content (Table 1). The only exception was the cheese ripened for 60 days in brine due to the decrease of PDM mass fraction between 40 and 60 days. Based on the results in Table 1, a significantly higher pH value of brined than of vacuum-sealed cheese was observed. This agrees with the NaCl and salt in moisture content in the cheese (Table 1). It seems that the pH of both cheese types intensively decreased after the first three days of ripening and then became almost constant. In fact, after an initial decrease the pH of vacuum-sealed cheese was constant after 20 days and then slightly but significantly (p<0.05) decreased, whereas the pH of the brined cheese was reduced during the first 20 days and then become constant. The observed changes in the pH values of vacuum-sealed cheese are in agreement with increasing acidity of these types of cheese. On the contrary, the acidity of brined cheese increased during the first 20 days, and then significantly (p<0.05) decreased. This can be explained as follows: changes in cheese pH during ripening are determined by the balance of the production of different organic acids (mainly lactic acid) that causes a drop in the pH and the buffering capacity of the cheese that resists this change in pH (*25*). Buffering capacity in cheese is related to proteins and inorganic constituents (weak acids, bases and metal ion complexes), especially inorganic P and Ca, which are not covalently attached to casein but are entrapped by the *para*-casein network (*26*). It can be assumed that proteolysis and ripening at low temperature (4 °C) induce the increase of these forms of Ca and P. Also, proteolysis occurred during the pretreatment of milk, the primary stage and the first 3 days of ripening inducing liberation of new amino and carboxylic groups which additionally contribute to high buffering capacity of cheese. Due to these facts, the retention of lactic acid increases the acidity but does not affect pH changes. The acidity of brined cheese started to decrease after 20 days when diffusion of low-molecular-mass compounds including nitrogen compounds, lactic and other organic acid occurred.

As expected, the vacuum-sealed cheese had a much lower salt mass fraction than the brined cheese. Regardless, both types can be considered as reduced-salt white cheese types since the observed values were lower than the values for Serbian white cheese types (3.48-5.43%) reported by Barac *et al*. (*18*).

### Nitrogen fractions and protein profiles

**Fig. 1:**
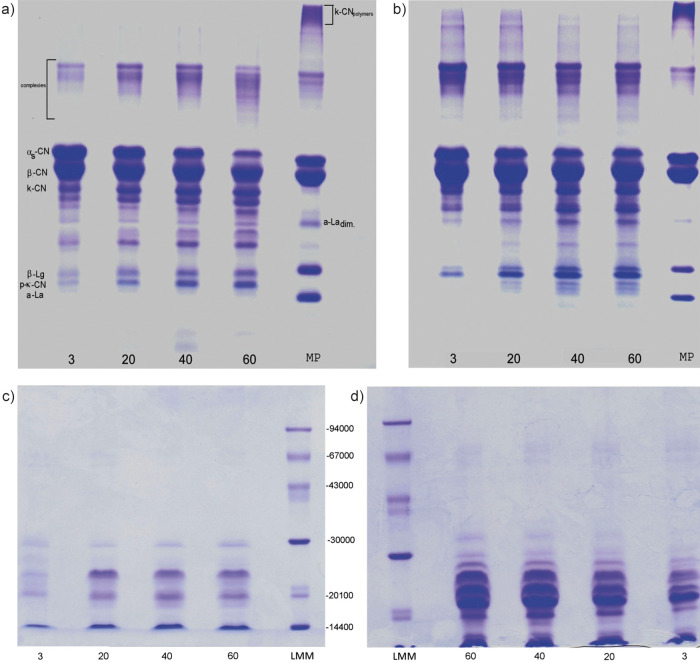
SDS-PAGE of: a and b) proteins of cheese ripened for 3, 20, 40 and 60 days in brine and vacuum, respectively, under non-reducing conditions, and c and d) of water-soluble fractions of cheese ripened for 3, 20, 40 and 60 days in brine and vacuum, respectively, under reducing conditions. MP=milk proteins, CN=casein, La=lactalbumin, Lg=lactoglobulin, La_dim_=dimmer of lactalbumin, LMM=low molecular mass standard

Table 2 shows the change of nitrogen mass fractions of white cheese during ripening and Fig. 1 the results of electrophoresis.

**Table 2:** The ripening parameters of white cheese ripened in brine or in a vacuum

(ripening)/day	Cheese ripened in a vacuum		
(*m*(WSN)/*m*(TN))/%	(*m*(PTA)/*m*(TN))/%	(*m*(TCA)/*m*(TN))/%
3	(5.07±0.09)^g^	(0.64±0.01)^c^	(4.5±0.2)^f,g^
20	(8.67±0.06)^f^	(0.64±0.03)^c^	(6.09±0.02)^c^
40	(13.23±0.08)^d^	(0.77±0.01)^b^	(7.02±0.01)^b^
60	(14.5±0.2)^c^	(0.96±0.02)^a^	(8.08±0.02)^a^
Cheese ripened in brine			
3	(10.3±0.5)^e^	(0.49±0.03)^d^	(4.4±0.1)^g^
20	(14.9±0.1)^c^	(0.50±0.01)^d^	(4.58±0.02)^f^
40	(20.18±0.04)^b^	(0.49±0.02)^d^	(5.14±0.01)^e^
60	(22.9±0.6)^a^	(0.78±0.03)^b^	(5.87±0.01)^d^

*m*(WSN)/*m*(TN)=mass of water-soluble nitrogen in the mass of total nitrogen, *m*(PTA)/*m*(TN)=mass of nitrogen soluble in 5% phosphotungistic acid in the mass of total nitrogen, *m*(TCA)/*m*(NT)=mass of nitrogen soluble in 12% trichloracetic acid in the mass of total nitrogen. Data within the same column marked with different lowercase letters are significantly different at p<0.05

The 3-day-old (fresh) cheese had significantly different mass fractions (in total nitrogen mass) of water-soluble nitrogen (WSN) and of nitrogen soluble in 5% phosphotungistic acid (PTA) and similar mass fractions of nitrogen soluble in 12% trichloroacetic acid (TCA) (Table 2). In fact, 3-day-old brined cheese had an almost 2-fold higher WSN mass fraction but significantly lower PTA mass fraction than their counterparts ripened in a vacuum.

The presence of soluble nitrogen fractions in fresh cheese could be a result of several processes, including those occurring during pretreatment of milk (thermolysis induced by high-heat treatment and possible proteolysis during 24 hours of kajmak separation), proteolysis during curd processing and during the initial 3 days of ripening. As the cheese samples are prepared from the same pretreated milk and according to an identical procedure, the differences observed in the soluble nitrogen compounds of 3-day-old cheese samples appear to be due to the choice of ripening environment and diffusion occurring in brined cheese. The WSN mass fraction is usually used as an index of cheese ripening and the higher values suggest more intensive initial proteolysis in brined cheese. The water soluble nitrogen mainly consists of the whey proteins, proteose-peptone, low-molecular-mass peptides derived from casein hydrolysis, free amino acids and weakly bound caseins (*27*). Since 3-day-old (fresh) cheese had low level of phosphotungstic acid-soluble nitrogen (PTA)-soluble nitrogenous substances (0.49 and 0.64% in brine and vacuum respectively) which reflect the content of free amino acids and small peptides (molecular mass lower than 600 Da) and similar values of the low-molecular-mass peptides (<15 000 Da), which are the major compounds of TCA-soluble nitrogenous substances (*14*), the higher WSN mass fraction in 3-day-old brined cheese can be attributed to the higher content of peptides with molecular mass higher than 15 000 Da. This observation was confirmed by SDS-PAGE analysis of WSN in 3-day-old cheese samples (Fig. 1b). The SDS-PAGE profile of 3-day-old brined cheese had more intensive bands of high-molecular-mass peptides (18-28 kDa), which are mainly products of residual rennet and residual heat-stable indigenous milk proteinases (*26*).

As expected, the mass fractions of soluble nitrogen (WSN, TCA and PTA) increased during ripening, but to different extents and with different trends. The WSN of both types of cheese increased continually and rapidly throughout the 60 days (Table 2) and reached a maximum of 14.54% (vacuum-sealed) and 22.88% (brined). These values are in accordance with those of 60-day-ripened (matured) low-fat white brined cheese (10-24%) reported by Romeih *et al.* (*28*). In contrast to WSN, the mass fractions of TCA and PTA increased at a much slower rate. In fact, in vacuum-sealed cheese, the TCA increased throughout the 60 days, whereas the increase of PTA was observed after 20 days. On the other hand, in the brined cheese, the increased values of TCA and PTA were observed after 3 and 40 days, respectively (Table 2). Relatively low mass fractions of TCA- and PTA-soluble nitrogen in both 60-day-old cheese types suggested slow proteolytic activity of starter and non-starter bacteria, probably due to low ripening temperature (4 °C) and the presence of heat-induced complexes less accessible to enzymes (*29*). Furthermore, the lower mass fraction of TCA- and PTA-soluble nitrogen in brined than in vacuum-sealed cheese can indicate slightly slower bacterial activity, which agrees with higher salt mass fraction in brined cheese (Table 1). Since salt mass fraction lower than 2.5% had no adverse effect on bacterial activity in the cheese (*30*), the lower mass fractions of low-molecular-mass nitrogen substances (TCA and PTA) in brined cheese should be attributed to their diffusion into brine rather than to slower bacterial activity. This is supported with the significant (p<0.05) decrease of dry matter content in the brined cheese and strong negative correlation (-0.955, -0.971; p<0.05) among TCA, PTA and dry matter content.

According to Fig. 1b, ripening conditions significantly affected SDS profiles of water-soluble peptides of both types of cheese. Ripening in brine at low temperature (4 °C) promotes the increase of the high- and medium-size molecular mass peptides (18-28 kDa). Besides these peptides, less intensive casein bands of α_s_-CN, β-CN and trace amount of polypeptides with molecular mass between 62 and 73 kDa could be observed. It is known that at a low temperature, due to the reduction in hydrophobic interactions, the dissociation of β-casein, and, to a lesser extent, dissociation of other individual caseins from the micellar structure occurred (*31*). In contrast to the brined cheese, in the WSN fractions of vacuum-sealed cheese only a band of β-CN was observed, which increased during ripening, while most of the detectable peptides were those with molecular mass of 14, 21 and 26 kDa (Fig. 1b). This suggested slower primary proteolysis in vacuum-sealed cheese, probably due to lower moisture and MNFS content and due to the lower pH, which is less favourable for plasmin activity than values detected in brined cheese (Table 1).

Hydrolysis of α_s_- and β-casein was expressed as a mass fraction (in %) of the corresponding mass of casein present on the third day of ripening (Table 3). As can be seen, the residual a_s_- and β-caseins in both cheese types continuously decreased during ripening. The rate of hydrolysis of the two caseins was different. In both cheese types the proteolysis of α_s_-casein was faster and more extensive than that of β-casein. This is attributed to the structure of the β-casein, which renders the molecule less accessible to enzymes (*32*). Also, it was shown that severe heat treatment additionally slows down β-casein degradation slightly and increases the rate of hydrolysis of α_s_-caseins (*33*), making these differences more pronounced. The breakdown of both caseins was significantly (p<0.05) faster in brined than in vacuum-sealed cheese. After 60 days, the residual α_s_- and β-casein in the brined cheese were 20.31 and 82.26%, respectively. At the same time, the residual α_s_- and β-casein in the vacuum-sealed cheese were about 65 and 92%, respectively (Table 3). The more extensive degradation of β-casein observed in brined than in vacuum-sealed cheese could be attributed to higher pH values in the brined cheese, which would enhance the activity of the indigenous milk proteinases (*34*).

**Table 3:** The change of residual α_s_-casein and β-casein during ripening of white cheese in brine and in a vacuum

*w*(residual casein)/%	*t*(ripening)/day			
3	20	40	60
Cheese ripened in brine			
α_s_-CN	100	77.41	61.08	20.31
β-CN	100	94.25	91.01	82.26
	Cheese ripened in a vacuum			
α_s_-CN	100	73.59	66.06	64.95
β-CN	100	96.71	94.82	92.07

The mass fractions of residual α_s_-CN and β-CN in ripened cheese were expressed as percentage of the mass fraction present in the extracts of 3-day-old cheese

It is interesting to note that the most intensive degradation of α_s_-casein in brined cheese was between the 40th and 60th day of ripening. During this period residual α_s_-casein declined from 61.08 to 20.31% of the α_s_-caseins present in the 3-day-old cheese. Since in this period also the most intensive loss of dry matter and protein in dry matter occurred (Table 1), it could be attributed mainly to the diffusion of α_s_-casein degradation products. This was completely consistent with the results of Romeih *et al.* (*28*).

The results presented in Fig. 1 and Table 2 clearly indicate higher proteolysis in the brined than vacuum-sealed cheese, mainly due to more intensive primary proteolysis. Our results contradict with those of Hayaloglu *et al.* (*35*) and Miloradovic *et al.* (*36*), who reported a higher proteolysis in the cheese ripened in a vacuum. Besides the different treatment of the milk, the observed disagreements could be attributed to the initial ripening period, during which the intensive processes, including proteolysis and diffusion in low-fat cheese occurred (*28*). Namely, in both reported studies, cheese was initially ripened (first 10 days) in brine and then transferred into vacuum plastic bags. In this study, after dry salting, cheese samples were continually ripened from the first day in brine or in a vacuum.

### Macro- and microelement profiles

Table 4 shows the mass fractions (g/100 g) of macro- (Ca, K, Mg, P and Na), and microelements (Fe, Zn, Cu and Mn) in the samples of reduced-fat cheese. Their contents in both cheese types were comparable with those in low-fat white brined cheese reported by Jaoude *et al*. (*37*). Contrary to previously reported data for Serbian full-fat white brined types of cheese (*18*) under the conditions used in this study, the presence of Pb, Cd, Li, B, Cr, Al and Ni was not detected. In both types of cheese Na, Ca and P dominated, whereas the most represented microelements were Zn and Fe. In general, ripening conditions differently affected the mass fractions of Ca, P, Na and Mg, whereas there was no significant influence on the mass fraction of K. Due to the dissolution of casein-bound Ca and P induced at low ripening temperature and their diffusion from the aqueous phase of cheese into the brine their mass fraction started to decrease from the 20 days of ripening. This phenomenon results in intensive binding of water if a small amount of sodium chloride is present, especially at pH=5.2–5.4 (*22*). This is in good agreement with the results reported in Table 1.

**Table 4:** The change of the mass fraction in lyophilized cheese of macro- and microelements during cheese ripening in brine and in a vacuum

				**Ca**		**Mg**		**Na**		**K**		**P**		**Fe**		**Cu**		**Zn**		**Mn**	
**Cheese**		*t*/day	*w*(lyophilized cheese)/(mg/100 g)										*w*(lyophilized cheese)/(µg/100 g)							
**Ripened in a vacuum**		3	(679±9)^a^		(25.1±0.5)^b^		(953±65)^c^		(123±10)^a^		(424±15)^a^		(358±11)^a^		(61.5±6.4)^a^		(2409±48)^d^		(14.0±0.5)^b^	
		20	(682±6)^a^		(25.0±1.1)^b^		(1111±97)^b,c^		(134±5) ^a^		(415±6)^a^		(355±14)^a^		(59.4±6.5)^a^		(2227±62)^f^		(9.8±0.6)^c^	
		40	(674±6)^a^		(24.6±0.5)^b^		(1023±63)^b,c^		(126±15)^a^		(395±10)^a^		(352±10)^a^		(69.5±2.1)^a^		(2800±17)^b^		(7.8±0.2)^d^	
		60	(684±11)^a^		(23.8±0.6)^b^		(958±44)^c^		(134±11)^a^		(401±8)^a^		(363±9)^a^		(67.2±2.3)^a^		(3068±25)^a^		(9.8±0.8)^c^	
**Ripened in brine**		3	(688±10)^a^		(30.8±0.4)^a^		(1350±15)^a^		(125±2)^a^		(410±11)^a^		(363±3)^a^		(12.5±2.5)^b^		(2605±16)^c^		(13.4±1.0)^b^	
		20	(680±17)^a^		(29.1±0.3)^a^		(1339±10)^a^		(130±14)^a^		(403± 8)^a^		(361±5)^a^		(12.4±1.9)^b^		(2304±33)^e^		(16.5±0.9)^a^	
		40	(626±15)^b^		(29.3±0.2)^a^		(1112±31)^b^		(126±14)^a^		(320±10)^b^		(350±4)^a^		(14.9±1.4)^b^		(2337±45)^e^		(14.2±0.2)^b^	
		60	(590±12)^c^		(18.1±0.3)^c^		(1106±12)^b^		(121±7)^a^		(300±3)^b^		(292±8)^b^		(14.1±1.7)^b^		(2287±30)^e^		(16.7±0.6)^a^	

Data within the same column marked with different lowercase letters are significantly different at p<0.05

The correlation analysis showed strong negative correlations (-0.977 and -0.952; p<0.05) between the time of ripening in brine and the mass fractions of Ca and P. Also, in these cheese samples a strong positive correlation (0.984; p<0.05) between the change of Ca and P mass fractions was observed. Ripening in brine longer than 40 days also reduced the mass fractions of Mg, Fe and Mn, whereas the mass fraction of Zn decreased after 20 days and then became constant. Contrary to the cheese ripened in brine, ripening in a vacuum had no significant (p<0.05) influence on the mass fractions of almost all detected macro- and microelements. The only exceptions were the mass fractions of Zn and Mn; after 60 days of ripening the mass fraction of Zn increased by 27.35%, whereas of Mn was reduced by approx. 30% (Table 4). Consequently, no significant correlation between the time of ripening and the mass fractions of individual minerals in this type of cheese was observed.

### Fatty acid profiles

Table 5 shows fatty acid profiles of white cheese ripened in brine and in a vacuum. As could be expected, the method used in kajmak production significantly affected fatty acid profiles of reduced-fat cheese types. Under experimental conditions used in this study, up to eleven fatty acids in 3-day-old brined and vacuum-sealed cheese were detected. Seven of them were saturated (SFA) and four were unsaturated (C4-C16), monounsaturated (MUFA) and polyunsaturated fatty acids (PUFA). As it is evident, fatty acid profiles of 3-day-old cheese samples were similar qualitatively. More precisely, the absence of oleic acid (C18:1n9c) from the brined cheese samples was the only qualitative difference from the 3-day-old vacuum-sealed cheese. However, more pronounced differences in the quantitative composition of fatty acid profiles of these samples is observable. In both 3-day-old cheese types, the SFAs dominated, but their mass fraction was higher (74.48%) in the 3-day-old reduced-fat brined cheese than in the vacuum-sealed cheese (71.51%). The most abundant saturated fatty acid in both 3-day-old cheese types was palmitic (C16); its average mass fraction in total fatty acid content of both types of 3-day-old cheese was 51.15 and 43.20%, respectively. The higher mass fraction of palmitic acid observed in the 3-day-old brined cheese samples can be attributed to the significantly lower mass fractions of short-chain (C4-C6) and medium-chain (C8) fatty acids, which represent only 2.64% of the identified fatty acids. In the vacuum-sealed cheese the mass fraction of these acids was 8.44%. These fatty acids are volatile and much more soluble than the long-chain fatty acids (*38*). Thus, the low mass fractions of C4-C8 fatty acids in the 3-day-old cheese ripened in brine is probably due to the diffusion of their free forms into the brine.

**Table 5:** Fatty acid profiles of white cheese ripened in brine and in a vacuum

*w*(fatty acid)/%	*t*(ripening)/day								
Cheese ripened in a vacuum					Cheese ripened in brine			
3	20	40	60	3		20	40	60
C4:0 butiric C6:0 caproic C8:0 caprilic C10:0 capric	(3.1±0.1)^b^	(4.09±0.08)^a^	n.d.	n.d.	(1.21±0.04)^c^		n.d.	n.d.	n.d.
	(3.22±0.01)^b^	(3.34±0.03) ^a^	n.d.	n.d.	(0.92±0.02)^c^		n.d.	n.d.	n.d.
	(2.10±0.05)^a^	(2.10±0.04)^a^	n.d.	n.d.	(0.51±0.03) ^b^		n.d.	n.d.	n.d.
	(3.74±0.01)^b^	(3.75±0.05)^b^	(3.72±0.06)^b^	(4.71±0.01)^a^	(3.35±0.08)^c^		n.d.	n.d.	n.d.
C12:0 lauric	(3.86±0.02)^d^	(4.02±0.01)^c^	(4.31±0.05)^b^	(4.83±0.02)^a^	(3.94±0.05)^c,d^		n.d.	n.d.	n.d.
C14:0 myristic	(12.27±0.08)^e^	(12.20±0.05)^e^	(16.70±0.10)^b^	(21.9±0.1)^a^	(13.40±0.09) ^d^		(14.8±0.5)^c^	(14.8±0.3)^c^	(16.0±1.0)^c^
C16:0 palmitic	(43.2±0.1)^h^	(41.40±0.09)^i^	(44.50±0.04)^g^	(43.60±0.06)^f^	(51.2±0.1)^d^		(65.0±0.2)^c^	(66.05±0.06)^b^	(66.60±0.08)^a^
C18:1n9c oleic	(2.20±0.09)^a^	(2.10±0.05)^a^	n.d.	n.d.	n.d.		n.d.	n.d.	n.d.
C18:1n9t elaidic	(12.92±0.07)^c^	(13.20±0.01)^b^	(16.81±0.05)^a^	(12.42±0.0)^d^	(12.51±0.02)^d^		n.d.	n.d.	n.d.
C18:2n6t linoleaidic	(11.76±0.08)^e^	(11.7±0.1)^e^	(13.04±0.03)^d^	(12.52±0.04)^d^	(11.91±0.09)^e^		(21.30±0.05)^a^	(19.22±0.02)^b^	(17.40±0.06)^c^
C18:3n6 γ-linoleic	(1.61±0.09)^b^	(2.20±0.01)^a^	n.d.	n.d.	(1.10±0.08)^c^		n.d.	n.d.	n.d.

n.d.=not detected. Data within the same row marked with different lowercase letters are significantly different at p<0.05

It is interesting to note that the major unsaturated fatty acids of both 3-day-old cheese types were elaidic (C18:1n9t) and linolelaidic (C18:2n6t) acid. The sums of their mass fractions in 3-day-old cheese were almost identical; they represented 24.42% (brined) and 24.68% (vacuum-sealed) of detected fatty acids. Both fatty acids are *trans*-fatty acids naturally present in the milk and cheese but at low amount. However, their mass fractions were higher than those reported for full-fat cheese (*18*), partly due to the separation of most of the commonly abundant milk fatty acids. Additionally, it is possible that under the heating conditions (90 °C, 60 min) used during kajmak production, part of naturally present unsaturated *cis-*fatty acids (such as oleic acid) are converted into their thermodynamically more stable *trans*-forms (*39*, *40*).

According to the results in Table 5, ripening in brine induces more intensive lipolytic changes than ripening in a vacuum. In cheese ripened in brine for 20 days, only three fatty acids (myristic, palmic and linoleaidic acid) were detected. In contrast, ripening in a vacuum longer than 20 days induces the loss of C4:0-C8:0, C18:1n9 and C18:3n6. Consequently, in the cheese ripened in a vacuum for 60 days, saturated fatty acids C10-C16 dominated.

### Antioxidant properties

The previous sections show that ripening in brine and in a vacuum differently affected protein, fatty acid and mineral profiles of reduced-fat cheese. It could be assumed that ripening in brine or in a vacuum also had different influence on their antioxidant properties. Therefore, the change of ABTS radical scavenging activity, iron(II) chelating activity and reducing power of the whole cheese during ripening was investigated. The obtained results are in Table 6.

**Table 6:** The change of antioxidant properties of reduced-fat cheese

*t*(ripening)/day	Fe(II) chelating ability/%	Reducing power (*A*_700 nm_)	ABTS inhibition/%	ABTS as BHT/(µg/mL)	
Cheese ripened in a vacuum				
3	(25.0±1.3)^e^	(0.372±0.008)^a^	(68.7±1.2)^d^	(109.01±6.1)^d,e^
20	(33.5±0.8)^d^	(0.37±0.02)^a^	(73.7±1.3)^c^	(97.6±6.4)^e^
40	(38.0±1.5)^c^	(0.385±0.007)^a^	(86.12±2.0)^a^	(140.7±3.8)^a^
60	(25.2±1.0)^e^	(0.37±0.01)^a^	(76.9±1.8)^c^	(123.0±3.5)^c^
Cheese ripened in brine				
3	(69.2±0.5)^a^	(0.35±0.01)^b^	(66.2±4.5)^d^	(102.4±8.6)^d,e^
20	(64.0±2.2)^b^	(0.37±0.01)^a^	(74.4±2.8)^c^	(118.1±5.4)^c,d^
40	(65.1±1.3)^b^	(0.40±0.02)^a^	(78.0±2.8)^b,c^	(125.0±5.5)^b,c^
60	(33.8±1.2)^d^	(0.261±0.009)^c^	(81.3±0.7)^b^	(131.4±1.3)^b^

ABTS=radical scavenging capacity, BHT=butylated hydroxytoluene. Data within the same column marked with different lowercase letters are significantly different at p<0.05

According to Table 6, 3-day-old cheese samples had similar ABTS radical scavenging activity, but significantly different iron(II) chelating activity and reducing power values. In fact, 3-day-old brined cheese had more than twice higher iron(II) chelating activity value and by 7.51% lower reducing power value than vacuum-sealed cheese. Iron(II) chelating activity of the cheese depends on the presence of aromatic and hydrophobic amino acids (especially the amount of histidine), phosphoserine residues, carboxylate groups (*41*) and the initial content of Fe^2+^ and other ions that can also chelate proteins. Since 3-day-old cheese samples had similar initial mass fractions of Fe^2+^ and macroelements (such as Ca, P and Mg) (Table 4), the higher values of iron(II) chelating activity in the brined cheese can be attributed to the more intensive initial proteolysis and more liberated carboxylate groups which can interact with iron ions.

The ripening in brine and in a vacuum differently affected the trend of iron(II) chelating activity and reducing power values. The iron(II) chelating activity of vacuum-sealed cheese increased during 40 days. During the same period, reducing power values of brined cheese slightly but significantly decreased. After that period, the ability of iron(II) chelating dropped sharply in both types of cheese. It reached values equal to those for 3-day-old vacuum-sealed cheese, while tthe values recorded in brined cheese were almost twice lower than the values of 3-day-old cheese, which is similar to those for 20-day-old vacuum-sealed cheese. The observed decrease of iron(II) chelating activity values agrees well with the previous results reported by Meira *et al.* (*42*). These authors suggested that the chelating activity of cheese proteins decreased as the hydrolysis degree increased, probably due to the degradation and/or diffusion of caseinophosphopeptides. This is supported by the loss of phosphorus shown in Table 4.

In contrast to iron(II) chelating activity, throughout 60 days of ripening of vacuum-sealed cheese, no significant changes of reducing power were observed, whereas in the brined cheese, it continually increased during 40 days and then dropped by 34.42%. However, the reducing power of both cheese types was similar or higher than the reducing power of traditional full-fat Serbian white brined cheese (0.156-0.296) reported by Barac *et al.* (*43*).

The ripening in brine and in a vacuum had a similar trend of ABTS radical scavenging activity. The radical scavenging activity of both cheese types slowly increased and reached maximum on the 40th day of ripening. After that period, the radical scavenging activity of brined cheese remained unchanged, whereas that of vacuum-sealed cheese decreased. The improved radical scavenging activity of different cheese varieties induced by ripening is documented (*4*, *42*) and associated with the increase of low-molecular-mass peptides, free amino acids and proteolytic changes that made the cheese gel network more able to scavenge free radicals. However, the decrease of radical scavenging activity of vacuum-sealed cheese samples observed between 40 and 60 days of ripening suggested that antioxidant peptides were susceptible to further proteolysis.

The correlation analysis showed that besides the time of ripening, the change of ABTS radical scavenging activity was significantly correlated only with water soluble nitrogen in dry matter (0.979, p<0.05; brined cheese) and protein in dry matter (-0.959, p<0.05; vacuum-sealed cheese). The absence of significant correlation between the ABTS radical scavenging activity and other investigated parameters has to be considered as a result of additive, synergistic or antagonistic interactions of caseins, residual whey proteins, peptides and free amino acids released during ripening and minor cheese constituents (such as phenols, vitamins, minerals, *etc*.) (*44*).

## CONCLUSIONS

The cheese ripening conditions (immersion in brine or in vacuum-sealed polyethylene bags) affected chemical composition, proteolysis, mineral content, fatty acid composition and antioxidant properties of reduced-fat white cheese prepared from the milk that remained after kajmak production. Dry matter, fat and protein contents were higher in the vacuum-ripened than in brined cheese, whereas a higher salt concentration was found in brined cheese. However, more intensive proteolysis was observed in brined cheese, mainly due to the higher degree of degradation of  α_s_-caseins. However, due to the absence of diffusion, the cheese ripened in vacuum bags had higher values of parameters related to secondary proteolysis. Also, vacuum-ripened cheese had higher content of Ca, P, Zn, Fe and Cu then brined cheese. After 60 days of ripening, in brined cheese myristic (C14:0) and palmitic (C16:0) saturated fatty acid dominated, whereas in vacuum ripened cheese, C10:0–C16:0 saturated fatty acids remained. Contrary to our predictions, due to more intensive proteolysis, 60-day-old cheese ripened in brine had more favourable ABTS radical scavenging and iron(II) chelating activities, but slightly lower reducing power than vacuum ripened cheese. The results of this study clearly indicate that ripening in a vacuum can be a useful process for obtaining nutritionally highly valuable cheese with reduced milk fat content.
